# Reminders make people adhere better to a self-help sleep intervention

**DOI:** 10.1007/s12553-016-0167-x

**Published:** 2016-12-23

**Authors:** Corine Horsch, Sandor Spruit, Jaap Lancee, Rogier van Eijk, Robbert Jan Beun, Mark Neerincx, Willem-Paul Brinkman

**Affiliations:** 1 Delft University of Technolgoy, Mekelweg 4, 2628 CD, Delft, The Netherlands; 2 Utrecht University, Utrecht, The Netherlands; 3 University of Amsterdam, Amsterdam, The Netherlands

**Keywords:** Adherence, Reminders, Sleep, Self-help, Compliance, Insomnia

## Abstract

The experiment presented in this paper investigated the effects of different kinds of reminders on adherence to automated parts of a cognitive behavioural therapy for insomnia (CBT-I) delivered via a mobile device. Previous studies report that computerized health interventions can be effective. However, treatment adherence is still an issue. Reminders are a simple technique that could improve adherence. A minimal intervention prototype in the realm of sleep treatment was developed to test the effects of reminders on adherence. Two prominent ways to determine the reminder-time are: a) ask users when they want to be reminded, and b) let an algorithm decide when to remind users. The prototype consisted of a sleep diary, a relaxation exercise and reminders. A within subject design was used in which the effect of reminders and two underlying principles were tested by 45 participants that all received the following three different conditions (in random order): a) event-based reminders b) time-based reminders c) no reminders. Both types of reminders improved adherence compared to no reminders. No differences were found between the two types of reminders. Opportunity and self-empowerment could partly mediate adherence to filling out the sleep diary, but not to the number of relaxation exercises conducted. Although the study focussed on CBT-I, we expect that designers of other computerized health interventions benefit from the tested opportunity and self-empowerment principles for reminders to improve adherence, as well.

## Introduction

Everyone forgets to do something now and then. The consequences of forgetting to do something depend on what was forgotten. Some of these memory failures can be fatal. Fogetting to buy milk is less problematic than forgetting to take a sleeping baby out of a soon to be hot car, or medical errors during surgery. The intention to do something in the future is formed in prospective memory [1]. Everyone suffers from prospective memory failures. In fact, Kliegel and Martin [2] state that 50–80% of everyday life forgetting is due to these prospective memory failures. Another study in the health domain found that most of the preventable mistakes were prospective memory failures [3]. Not only medical professionals suffer from prospective memory failures, also patients suffer from it. For example, people forget to take their pills. Forgetfulness, or prospective memory failure, is one of the main reasons [4–6].

Several models attempt to explain how prospective memory works e.g., the preparatory attentional and memory theory, the reflexive-associative theory, and the multi-process model [1]. The latter two theories include cues. The idea is that an intended action is associated with a cue. When that cue occurs the intended action is remembered automatically. Reminders provided for example by a smartphone can serve as these cues and might play an important role in performing targeted behaviour.

Reminders have been used in various domains in different forms for a long time. Ranging from tying a string around your finger, self-written notes, to reminders set on PDAs, watches, and smartphones. Fogg describes three types of triggers in his behavioural model [7]. He distinguishes sparks, facilitators, and signals. A spark is a cue that enhances motivation. There are three core motivators that sparks can use: pleasure-pain, hope-fear, and social acceptance-rejection. A facilitator is a cue that makes it easier to exhibit a certain type of behaviour (enhances ability). A signal is a simple reminder used in cases where both motivation and ability are high. Another distinction in reminders can be made based on the trigger method utilized. Various trigger methods are time-based, event-based, and location-based. An example of a time-based action is taking cookies out of the oven in 20 min, an example of an event-based action is bringing up an issue during the next meeting, and a location-based action is throwing a letter in a mailbox when passing by. Prospective memory also makes use of these type of triggers, and research has shown that people perform better at event-based intentions than at time-based intentions [8]. Especially if target behaviour has to be performed at a specific time, people could benefit from a reminder system.

Furthermore, interruptibility has been studied extensively (e.g. [9–11]). Traditionally, task complexity, task duration, and the moment of interruption has been identified as determining factors for the appropriateness of an interruption [12]. Recently, mobile interruptibility studies shifted the focus to the moment of interruption. Mobile studies have shown that smartphone notifications can have inappropriate timing [13, 14]. In mobile interruptibility studies context is often mentioned as the determining factor for the appropriateness of an interruption. Context, however, is a comprehensive concept that is used differently in studies. For example, Ho and Intille [11] measure physical activity and appropriateness of interruptions, whereas Pielot and colleagues [15] use phone usage data to infer interruptibility. Independent of the definition of context that is used, all studies acknowledge the importance of appropriate timing.

There is substantial evidence that computerized health interventions can be effective [16–18]. However, adherence remains a challenge. Compared to more traditional treatments, computerized interventions can be experienced as less binding, therefore it is easy to drop-out [19]. Since, the efficacy of treatment is partly determined by adherence [20–23] it is crucial to optimize adherence [24]. One of the reasons why people do not adhere to health interventions is forgetfulness [22, 25]. Reminders are a simple technique that could help solving this particular problem of forgetfulness [25]. For example, earlier studies in the health domain have shown that mobile text reminders increase show-up rates for gastrointestinal endoscopy [26], for breast cancer screening [27], and sunscreen use [28]. Another example, regarding an app with notifications, showed an increase from 12% to 63% in logging food intake on a mobile phone when reminders were given compared to the absence of reminders [29]. Moreover, a systematic review about reminders in cell phone interventions found a difference in 20 of the 25 studies between the intervention and control group [30]. This indicates that reminders improve adherence and the outcome of interventions.

Previous research suggests that reminders can be effective, to our knowledge, however, barely any empirical work has been done regarding the underlying principles that explain why these reminders work. This paper discusses and tests two prominent reminder approaches: time-based reminders and reminders inspired by the interruptibility literature. The time-based reminders are self-set reminders in which the user can choose the time. The other reminders are automatic event-based reminders, inspired by the Capability-Opportunity-Motivation Behaviour (COM-B) model [31]. Here the system detects opportune moments and send a reminder.

A domain that can benefit from effective reminders is mobile insomnia treatment. People who suffer from insomnia have difficulties initiating or maintaining sleep [32]. Having insomnia leads to personal suffering, like a reduced quality of life, and societal costs, like reduced productivity [33]. Studies estimate that about 10% of the adults suffer from insomnia [34]. Cognitive-Behaviour Therapy for Insomnia (CBT-I) is the treatment of choice for this disorder and fairly standardized in protocols [35]. CBT-I consists of several exercises that requires behaviour changes, however, adherence to CBT-I remains a problem [22, 36]. For instance, a daily sleep diary that helps people to become aware of their sleep behaviour and monitor progress is easily missed. Reminding people to do their exercises could be beneficial and provides opportunity to test the effect and underlying principles of reminders.

## Reminder design and hypotheses

Earlier work has shown that reminders probably work, but it might depend on the domain, the patient demographics, psychosocial and behavioural characteristics, etc. [37, 38] Therefore, the first step is investigating if reminders in an sleep intervention domain, delivered via a smartphone are effective. So, hypothesis 1 is:

- ***H1: Reminders increase adherence compared to no reminders.*** When people are reminded to do something they will do it more often, compared to situations in which they are not reminded to do it. There was no hypothesis regarding an adherence difference between the two types of reminder.

**Fig. 1:**
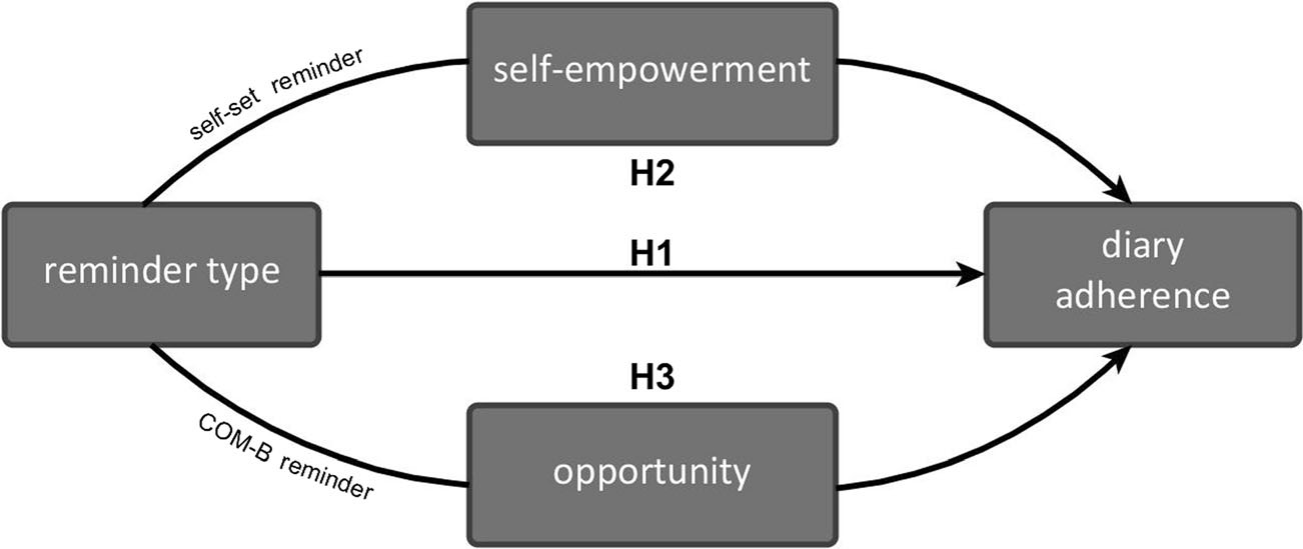
The three hypothesized relationship between the type of reminder, the explaining concepts and adherence

Figure 1 depicts this and the following hypotheses regarding the effect of reminders and their underlying mechanisms.

### Self-set reminder

Having users set the reminder times themselves (instead of automatically set reminders) might be an adequate mechanism, because users know best when they have time to perform an activity. Several psychological principles support and explain why self-set reminders increase adherence. Firstly, the self-determination theory [39] states that supporting autonomy, competence, and relatedness increases people’s motivation and performance. By giving users the control to set the reminder times, their autonomy is supported and thereby their motivation and performance increases. Secondly, the consistency principle [40] states that humans want to be consistent in their attitudes, beliefs, perceptions, and behaviours and they will change any of these when inconsistency is discovered. This suggests that, when people set the reminder times themselves, they are more likely to follow-up on those reminders, because they want to be consistent. Thirdly, predictability could be seen as a basic human motive. From an evolutionary viewpoint, higher predictability of (dangerous) events gives a better chance of survival. Campbell and Tesser [41] construct the predictability motive from the human desire for certainty, the need for an understandable world, and the need to be able to predict the environment. In line with this predictability motive, reminders will be perceived more positive when arriving at predictable times, and adherence will benefit from this positive attitude.

In conclusion, self-set reminders could help to improve adherence because of three underlying principles. First, users would probably feel more in control and therefore respond more positive to the reminders. Second, people might feel committed to their self-set reminders, which would also elicit more positive responses. Third, the reminders are more predictable when the users set the times themselves, and this should also improve the response to reminders. In summary, these self-set reminders should increase the sense of self-empowerment, and therefore increase treatment adherence.

- ***H2: If self-set reminders are given, self-empowerment mediates adherence.*** When people receive reminders at times they set themselves, they feel more empowered. In which self-empowerment includes, perceived control, commitment and predictability. So, it was hypothesised that self-set reminders increase perceived self-empowerment, and that perceived self-empowerment was associated with their adherence. Therefore, self-empowerment could partly explain adherence, when self-set reminders were given.

### COM-B reminder

Reminders can also be triggered automatically. The Capability-Opportunity-Motivation-Behaviour (COM-B) model states that the possibility that people exhibit a behaviour depends on the capability of a person, the opportunity, and their motivation to exhibit that behaviour [31]. Opportunity in this context means the circumstances that allow someone to exhibit the targeted behaviour. For example, taking the stairs, instead of the elevator, is only possible when there are stairs (opportunity). Besides, it is easier to climb stairs when they are located in front of the entrance (opportune location), or when colleagues take the stairs (social opportunity). The model suggests that if people are capable and motivated to exhibit the targeted behaviour, a reminder at an opportune moment, improves the change a person will exhibit this behaviour. This reminder design is in line with earlier work regarding interruptibility.

- ***H3: If COM-B reminders are given, opportunity mediates adherence.*** It was hypothesized that when people receive these automatic reminders, it was an opportune moment to perform the targeted behaviour. Therefore, it was expected that opportuneness was associated with people’s adherence, thereby partly explaining an increase in adherence for COM-B reminders.

## Method

### Experimental design

This field experiment had a within-subjects design with 45 participants who were exposed to three conditions during a total time of three weeks. In one condition participants received no reminders to perform targeted behaviour, in the other condition participants set the reminders themselves, and the last condition consisted of automatic COM-B reminders. The order of the three conditions was counter-balanced across the participants. The study was approved by Human Research Ethics Committee of Delft University of Technology.

### The intervention system

An app for people suffering from insomnia was developed to test the two types of reminders. Since most people always carry their phones with them, smartphones were suitable for reminders. The app contained a sleep diary, a relaxation exercise, sleep overview graphs, and reminders. The two different activities were chosen to measure adherence because they have different properties. For example, it was probably easier for people to spend 1 min, which is the approximated time for filling in the diary, than 7 min, which is the length of the relaxation exercise.

**Fig. 2:**
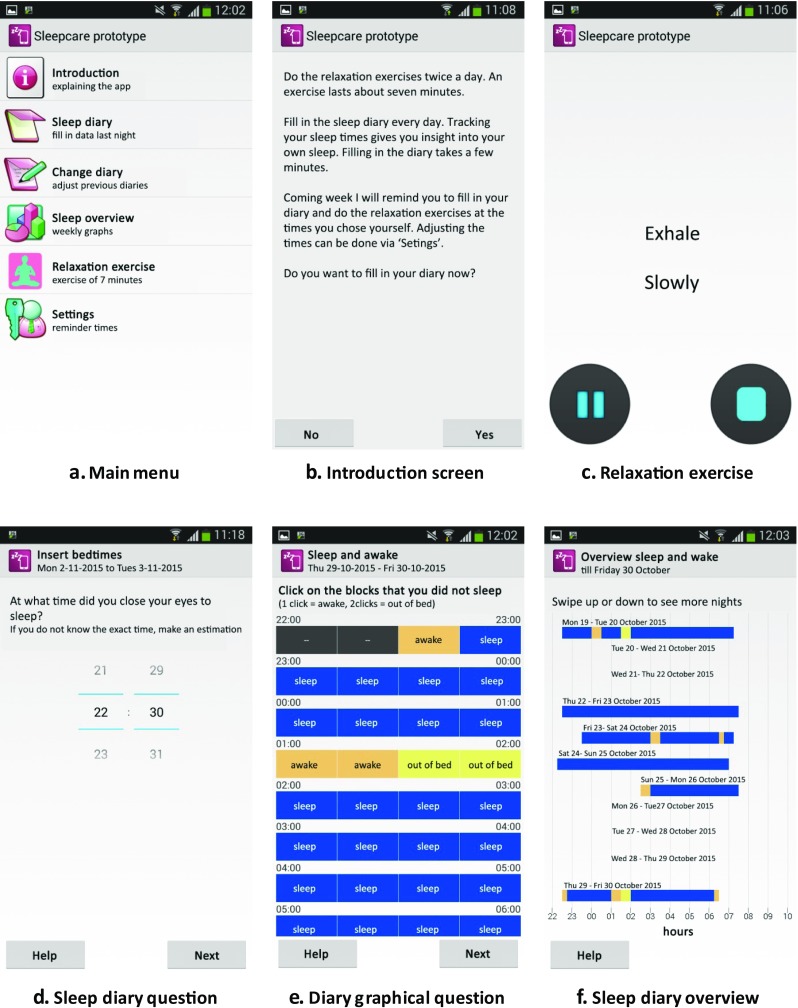
Screenshots of the Sleepcare app translated from Dutch

Navigation in the app was done using the main menu (Fig. 2a), containing all the elements of the app. In the introduction screen (Fig. 2b) a short explanation of the relaxation exercise and the sleep diary was given, as well as information about the reminders. Furthermore, the app contained a progressive muscle relaxation exercise (Fig. 2c). The instructions were both visual on the screen in text and simultaneously audible. Moreover, the app contained a sleep diary consisting of four screens each with one question on it, respectively: (1) what time did you go to sleep?, (2) what time did you get up?, (3) indicate when you were awake, (4) which score would you give your sleep? (Fig. 2d and e). Via a different menu-item users could correct mistakes in their sleep diaries. An overview of users’ tracked sleep was shown in a graphical overview (Fig. 2f). The last menu-item was settings. Here, people could change the times of the reminders in the self-set condition. Participants chose the time for three daily reminders: one reminder for filling out their sleep diary, and two others for doing the relaxation exercise.

For the automated reminders, we assumed that if people download an app, they were able and motivated to use the app. Leaving one requirement to exhibit a behaviour to fulfil, namely opportunity. The opportunities to exhibit the targeted behaviour were automatically detected by the smartphone based on the smartphone usage. The sleep diary needs to be filled out as closely as possible to waking up. So, users received a reminder for filling in the diary the first time they turned on their phone in the morning. Reminders for the relaxation exercises were generated when people: a) were at the same physical place for more than one hour, since it was regarded appropriate to take a break when sitting still for some time; b) ended a phone call, and thus were already distracted from another (important) task; or c) used another app,^1^ and thus were already using their phone. Reminders were of course not generated when users already filled in their diary or did the relaxation exercise, or were occupied with that activity. Moreover, the time between two reminders was at least 30 min.

### Procedure

Participants were recruited via social media, online advertisements, the website www.ikgalekkerslapen.nl, and in university lectures. After giving online consent and completing the first questionnaire, participants were enabled to download the app. The Sleepcare app ran on Android OS versions from 2.3 and higher. Participants used their own smartphone with Internet connection for this experiment. A short introduction text about the app was shown the first time participants opened the app (Fig. 2b). The participants were instructed to fill out the sleep diary every day, and do the relaxation exercise twice a day. However, they were free to use the app in whatever way they wanted. After every week, participants got an e-mail with a link to that week’s questionnaire and to inform them about the change of condition (within-subjects). Within two weeks after finishing the whole experiment participants were contacted for a semi-structured telephone interview.

People who gave informed consent, but did not download the app, received an e-mail with the question why they did not proceed with the experiment or app. Also participants from whom only a few days of data was received, got a similar e-mail to gain insight in reasons for non-adherence.

### Measurements

#### Primary outcome and mediation measures

During the experiment, adherence was measured by logging how often the diary was filled out (0–7 days), and how often the relaxation exercise was performed. These were the primary outcome measures. The mediation measures were the level of perceived self-empowerment and the suitability of the timing of the reminders (opportunity). Both mediation measures were measured using a questionnaire specially designed for this study consisting of 7-point Likert scale statements (Appendix 4). Both concepts were measured in the two reminder conditions to examine if these concepts were able to explain adherence rates. Furthermore, motivation and ability were measured to check the assumptions of the COM-B model. The COM-B model states that the possibility that people exhibit a behaviour depends on the opportunity, the person’s motivation to exhibit that behaviour, and the capability of a person to exhibit that behaviour [31]. Opportunity was measured as one of the mediators and was expected to vary across the study. Motivation and capability, on the other hand, were expected to be constant during the experiment, so they would not influence adherence. In order to check this assumption motivation was measured using the Situational Motivation Scale (SIMS) [42], consisting of Intrinsic Motivation (IM), Identified Regulation (IR), External Regulation (ER), Amotivation (AM). And ability was measured in three categories: ability to use a smartphone (AUS), ability to use the diary (AUD), and ability to use the relaxation exercise (AUR), using 7-point Likert scale statements (e.g. “*I know how I can respond to notifications on my smartphone*”) (Appendix 2 and 5).

#### Exploratory measures

In order to perform more detailed, exploratory analyses that fall outside the main focus of the paper, the following measurements were taken. Firstly, an earlier study showed that objective (logged) and subjective (self-reported) adherence deviate from each other [22]. Therefore, participants’ own estimation about their adherence was explored. This subjective adherence (SA) was measured by the questions ‘How often did you fill in the sleep diary last week?’, and ‘How often did you do the relaxation exercise last week?’ (Appendix 3). Because both objective and subjective adherence were measured in this study these two concepts could be compared to each other and the reliability of participants’ own estimation about their behaviour and adherence could be derived.

Behavioural intention (BI), locus of control (LoC), irritation (Irr) and appreciation, and easiness to use the app in daily life (ETI) were measured to be able to examine possible associations between these variables and adherence rates. The theory of planned behaviour states that behavioural intention predicts behaviour [43]. Therefore, behavioural intention (BI) was measured using six questions (e.g. “I will follow the instructions/advice from the app”). Locus of control (LoC) was measured via a 18-item Dutch questionnaire [44]. A higher internal locus of control has been found to influence diary adherence in an online lifestyle diary [45]. Irritation and appreciation were respectively measured with four 7-point Likert scale statements, and assigning a grade between 1 and 10 for the different reminders and app components. Reminders that irritate people because they are disruptive, or reminders that are not appreciated most likely decrease adhere [13, 14, 46].

Similarly, if an activity is hard to integrate in people’s daily life, the probability that people will perform the activity decreases, since people’s behaviour are affected by the principle of least effort [47]. Therefore, easiness to use was measured with six 7-point Likert scale statements. Furthermore, the Unified Theory of Acceptance and Use of Technology (UTAUT) measures technology acceptance and relates acceptance to usage [48], thereby possibly explaining adherence. Moreover, to investigate the possible effect of this minimal prototype on sleep the Insomnia Severity Index (ISI) was used [49]. See the appendices for more details about these measures.

At the end of the experiment participants were contacted for a semi-structured telephone interview to explore their reasons for (none-)adherence. The subjects of the questions were: why people used the app, what their opinion was about the app and the separate parts of the app, how people used the app, if they noticed any effect (on sleep or in other ways), if there were any irritations, and if people had ideas for improvements or additions (See appendix 6 for the used interview guide).

### Participants

**Fig. 3:**
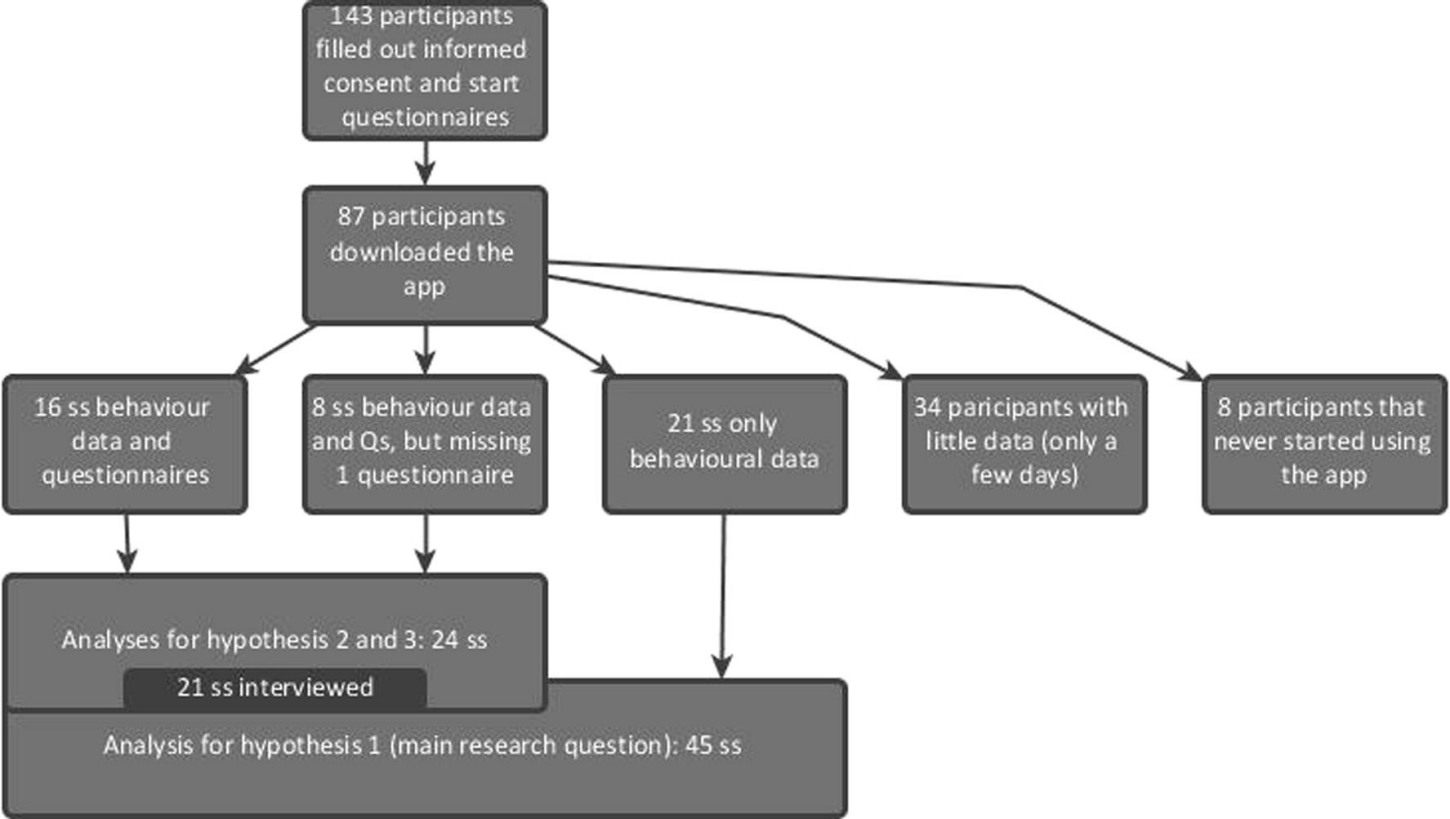
Flow diagram of participants in the experiment. Qs = Questionnaires

In total there were 45 participants who used the app for three weeks (Fig. 3), 30 females and 15 males. The average age was 35 years (*SD* = 14). Their average ISI score was 13.5 (*SD* = 6.6), which is above the score of 10 (*t* = 2.60, *p* < .05) that is used as a cut-off for clinical levels of insomnia [32]. The self-reported average of the ability to use a smartphone was 5.7 (*SD* = 1.3) on a scale from 1 to 7, which is an average rating on the positive side of the scale (*t* = 6.26, *p* < .01).

Although no strong conclusions can be drawn from a relatively arbitrary comparison with of the middle of the scale, participants seem positive about their smartphone abilities. Intention to use the app was 6.5 (*SD* = 0.7) on a score from 1 to 7, which also is an average rating on the positive side of the scale (*t* = 17.25 , *p* < .01). These scores suggested an adequate level of ability and motivation. As shown in Figs. 3, 143 participants filled out the online informed consent form and the pre-measurements and 87 participants downloaded the app. From all those 87 participants automatic log files were received which describe their app usage (behaviour data). However, not everyone filled in the weekly online questionnaires. Sixteen participants filled in all three questionnaires, whereas 8 participants only filled in two questionnaires. The data of these 24 participants was used for the analyses regarding hypotheses two and three. Twenty-one participants filled in less than two questionnaires. Their logged behavioural data, together with the data from the 24 previously mentioned participants, was used for analysis regarding hypothesis one. Thirty-four participants only used the app for a few days, and 8 participants did not use the app at all. As a consequence their data was not used for the analyses.

### Statistical analyses and data preparation

For data management and analyses, SPSS version 22 was used. To test hypothesis 1 Friendman’s ANOVA tests were performed. Furthermore, posthoc Wilcoxon’s test were done to investigate the differences between the separate conditions. Hypotheses 2 and 3 were tested by repeated measures mediation analyses. The analyses were done in line with the method described by Judd et al. [50]. The first step in this method is to test the overall treatment effects for the dependent variable (adherence), as well as for the mediators (self-empowerment and opportunity). This means testing for a difference in adherence between the three conditions using Friedman’s ANOVAs (the same as used to test hypothesis 1), and testing for a difference in self-empowerment between the two reminder conditions using Wilcoxon’s test, and testing for a difference in opportunity between the two reminder conditions using Wilcoxon’s test. Although the original procedure suggests not to proceed with mediation analysis if no differences are found in this first step, Zhao et al. [51] claim this is too strict. Zhao explains that mediation can occur even when there is no total effect of independent variable on the dependent variable, so the guidelines of Zhao et al. [51] were followed. The second step was estimating the regression equation given by Judd et al. [50] (Box 1) that indicates mediator and moderator effects. Four different regression equations were used with a bootstrapping samples of 1000. Each analysis took as dependent measure either adherence to the diary or relaxation exercises. Self-empowerment (SE) score or Opportunity (Opp) score were included as mediator in these analyses.

**Table:** Box 1 Regression equation belonging to repeated measure mediation (Judd et al., 2001)

$$ {Y}_{Di}=\left({\delta}_2-{\delta}_1\right)+{\scriptscriptstyle \frac{1}{2}}\left({\delta}_{22}-{\delta}_{11}\right){X}_{Si}+{\scriptscriptstyle \frac{1}{2}}\left({\delta}_{22}+{\delta}_{11}\right){X}_{Di} $$ (1)
*Y*_*Di*_: Difference in diary/relaxation adherence between COM-B and Self-set condition
*Y*_*Si*_: Sum of self empowerment/opportunity score in COM-B and Self-set condition
*Y*_*Di*_:Difference between self empowerment/opportunity score in COM-B and Self-set condition
Mediation is suggested if the 3^rd^ coeffecient $\left({\scriptscriptstyle \frac{1}{2}}\left({\delta}_{22}+{\delta}_{11}\right)\right)$ is significanct.
Moderation is suggested if the 2^nd^ coeffecient $\left({\scriptscriptstyle \frac{1}{2}}\left({\delta}_{22}-{\delta}_{11}\right)\right)$ is significanct.

For testing hypothesis 1 the behavioural data collected via the app of 45 participants was used (Fig. 3). For testing hypotheses 2 and 3 the questionnaire data was needed. We hypothesised that participants just forgot to fill in a weekly questionnaire unrelated to the week and condition, so that the data was missing completely at random. Little’s MCAR test confirmed this assumption (Chi-square < 0.001, *df* = 1692, *p* > 0.99). Therefore, hypotheses 2 and 3 were tested using the data of the 16 complete datasets plus 8 datasets which missed one weekly questionnaire (amount of missing values: 388/5064 = 8% data points). This missing data was filled in using the expectation maximization method [52] using all variables except the demographics.

#### Exploratory analyses

To explore a possible effect of the app on insomnia a *t*-test with the ISI scores measured before and after the experiment was performed. Furthermore, Cronbach’s alphas were calculated for the concepts that were measured with multiple questions, such as opportunity (Opp), self-empowerment, and irritation (Irr). Items that affected Cronbach’s alphas negatively were deleted resulting in Cronbach’s alphas ranging from .43 to .99 (see appendices). The average scores for each concept were calculated with the remaining items. Next, Friedman’s and Wilcoxon’s tests were performed to test differences between conditions for repeated measures (Table 3). Besides, correlations between adherence and repeatedly measured variables were calculated using the procedure of Bland and Altman [53].

The interviews were analysed in line with the method of thematic analysis [54]. The first author (CH) familiarized herself with the data by conducting the interviews and transcribing the audio files. The data of all the participants was then organized per question, and codes were added to the answers. Lastly, the codes were grouped together in themes and a brief summary of the general gist was written.

## Results

### Hypothesis 1 – the effect of reminders

The results confirmed hypothesis 1. In the no reminder condition a median of 4 (IQR = 6) filled in diaries per week was found, and a median of 0 (IQR = 6) performed relaxation exercises were done. In the self-set reminder condition in median of 6 (IQR = 2) was found for the diaries, and a median of 1 (IQR = 3) for the relaxation exercise. In the COM-B condition a median of 7 (IQR = 3) filled in diaries was found, and a median of 1 (IQR = 5) for the relaxation exercises (Table 1). Friedman’s ANOVA’s showed differences between the conditions for the number of diaries filled in (*χ* ^*2*^(2) = 14.63, *p* = .001), and for the number of relaxation exercises done (*χ* ^*2*^(2) = 9.04, *p* = .011). To further investigate the differences, Wilcoxon tests were performed in which the *p*-values were tested against Bonferroni corrected α-level of .0167. These analyses showed a difference between the condition without reminders and the conditions with reminders, but no differences were found between the two reminder conditions (Table 1).

**Table 1:** Wilcoxon tests with Bonferroni correction showing differences between no reminder and a reminder, but not between the two types of reminders

Number of filled in diaries:	
No Reminder (*Mdn* = 4) vs. Self-Set (*Mdn* = 6)	*T* = 95.5, *p* = .002, *r* = −.27
No Reminder (*Mdn* = 4) vs. COM-B (*Mdn* = 7)	*T* = 67.0, *p* < .001, *r* = −.31
Self-Set (*Mdn* = 6) vs. COM-B (*Mdn* = 7)	*T* = 152.0, *p* = .78, *r* = −.02
Number relaxation exercises done:	
No Reminder (*Mdn* = 0) vs. Self-Set (*Mdn* = 1)	*T* = 42.5, *p* = .001, *r* = −.28
No Reminder (*Mdn* = 0) vs. COM-B (*Mdn* = 1)	*T* = 84.0, *p* = .011, *r* = −.22
Self-Set (*Mdn* = 1) vs. COM-B (*Mdn* = 1)	*T* = 192.5, *p* = .81, *r* = −.02

### Hypothesis 2 and hypothesis 3 – mediation

Hypotheses two and three were confirmed by repeated measures mediation analyses. The first step of Judd’s [50] procedure contains difference tests for the dependent variable and the mediators. The analyses done for hypothesis 1 already showed a difference in adherence (dependent variable) between the two conditions. The differences for the mediators are shown in Table 3. Self-empowerment scores were on average higher in the self-set condition than in the COM-B condition (Table 3). The opportunity scores were on average higher in the self-set condition than in the COM-B condition for the relaxation exercise, for the diary this difference was not significant (Table 3). The second step of the repeated measures mediation analyses contains the four regression equations (Box 2). In the mediation analysis on the number of diary entries for self-empowerment (SE) we found a significant positive mediation effect, whereby increase in self-empowerment was associated with increase in adherence. The second mediation analysis on diary adherence included opportunity (Opp) score as mediator. The analysis found also a significant mediation effect. Here an increase in opportunity score coincides with increase in number of diary entries filled out. Two similar mediation analyses were conducted on the adherence of the relaxation exercise. No significant mediation effects were found.

The COM-B model assumes that participants are able and motivated to perform the targeted behaviour. To test these assumptions the scores for ability and motivation are reported. On a seven-point scale (values 1–7) general ability (AUS) to use a smartphone was 5.7, ability to fill in the diary was 6.6, and ability to do the relaxation exercise was 5.5. These high values seem to confirm our assumption about participants’ capability. The identified regulation (IR) scores were the highest among the motivation scores, which suggests that participants were mostly using the app, because they wanted to use the app. Furthermore, the average amotivation (AM) scores were low. These values again seem to confirm our assumption about participants’ motivation.

**Table:** Box 2 Regression equations belonging to the repeated mediation analyses

Regression functions	
Diary adherence (diary self-empowerment):	*Y*_*Di*_ = − 2.39 + 0.14*X*_*Si*_ + 0.39 ^*^ *X*_*Di*_
Diary adherence (diary opportunity):	*Y*_*Di*_ = − 3.17 + 0.32*X*_*Si*_ + 0.48 ^*^^*^ *X*_*Di*_
Relaxation adherence (relaxation self-empowerment):	*Y*_*Di*_ = 0.31 − 0.05*X*_*Si*_ − 0.24*X*_*Di*_
Relaxation adherence (relaxation opportunity):	*Y*_*Di*_ = 2.22 − 0.35*X*_*Si*_ − 0.25*X*_*Di*_
	^*^ *p* <.001, 95% CI 0.14–0.59, mediation
	^**^ *p* = 0.45, 95% CI 0.03–0.95, mediation

### Exploratory analyses

Table 2 shows that four variables were associated with adherence. Diary adherence was correlated to behavioural intention and ability to use the diary (AUD). Relaxation adherence was correlated to the appreciation for the relaxation exercise and the general ability to use a smartphone (AUS). As can be seen the UTAUT concepts were not found to be correlated neither to diary nor relaxation adherence. There was a significant difference in sleep severity (ISI) before (*M* = 13.50, *SD* = 6.60) and after intervention (*M* = 11.28, *SD* = 6.03) *t*(23) = 2.74, *p* = .012, *r* = .50 . Although people suffer less from insomnia after using the app, it was not a clinically meaningful difference, which is set on a 6-point reduction [55].

**Table 2:** Means and standard deviations of the measurements, and their correlations with behavioural adherence

Measures (n = 24)	Mean (SD)	Pearson’s Correlations			Mean (SD)	Pearson’s Correlations	
		Diary	Relax			Diary	Relax
Pre-measures				UTAUT-concepts (post)			
AUS	5.71 (1.28)	–.20	–.43*	Utility	5.03 (1.26)	.37	.20
Behavioural intention	6.48 (0.70)	.62**	.30	Effort	6.41 (0.58)	.39	.33
Locus of Control^a^	7.08 (3.44)	.10	.05	Social influence	2.30 (1.45)	–.15	–.13
Insomnia Severity	13.50 (6.60)	.18	-.15	Facilitating conditions	4.93 (1.48)	.21	.16
				Attitude	5.91 (0.81)	.09	.31
*Post-measures*				Self-efficacy	6.73 (0.53)	.25	–.21
Appreciation diary^b^	8.09 (1.77)	.39	-	Anxiety	1.95 (1.12)	.27	.19
Appreciation relax^b^	5.74 (2.36)	-	.53**	Trust	5.43 (1.19)	.36	.16
Appreciation app^b^	7.31 (1.55)	.35	.32	Behavioural intention	5.87 (0.81)	.27	.10
AUD	6.63 (0.76)	.59**	-				
AUR	5.46 (1.64)	-	–.04				
Insomnia Severity	11.28 (6.03)	–.01	–.21				

*AUS* Ability to Use a Smartphone, *AUD* Ability to Use the Diary, *AUR* Ability to Use the Relaxation exercise

^a^scale ranges from 0 to 18, higher scores mean higher external locus of control

^b^grade given by the participant for the indicated component measured on a scale from 1 to 10

* *p* < .05, ** *p* < .01

Table 3 shows the variables that were measured repeatedly. Self-empowerment (SE) differed over the conditions for both the diary and the relaxation exercise. The opportunity score (Opp) only differed for the relaxation exercise, not for the diary. In addition, there was a correlation between self-empowerment and diary adherence, and between opportunity and diary adherence. These correlations were not found for the relaxation exercise.

**Table 3:** Means and standard deviations of the repeated measures, the difference between conditions for these measures, and their correlations across conditions with behavioural adherence

	Diary				Relaxation			
	NR	Self-Set	COM-B^c^	r	NR	Self-Set	COM-B^c^	r
Measures								
SE		4.72 (0.86)	3.84 (0.78)**	.50*		4.36 (1.06)	3.07 (0.89)**	–.18
Opp		5.46 (1.17)	5.33 (1.46)	.45*		3.70 (1.50)	2.98 (1.59)*	.11
SA	6.21 (1.76)	6.56 (1.01)	6.26 (1.59)	.67**	6.65 (5.31)	7.63 (5.09)	6.44 (4.14)	.82*
Sat	5.89 (1.62)	6.22 (1.32)	6.19 (1.43)	.57**	3.84 (2.14)	4.18 (1.99)	3.48 (1.97)*	.18
ETI	5.65 (1.24)	5.67 (1.10)	5.94 (1.08)	.33*	3.79 (1.81)	3.96 (1.49)	3.45 (1.59)	.00
Irr^a^		5.31 (1.01)	5.44 (1.31)	.62**		5.12 (1.36)	4.23 (1.79)*	–.13
IM	3.88 (1.07)	4.39 (1.28)	4.52 (1.20)**	.30*	3.46 (1.01)	3.62 (1.12)	3.57 (1.20)	.28*
IR	5.19 (1.17)	5.22 (1.06)	5.36 (0.99)	.25	4.92 (1.37)	5.07 (1.27)	5.07 (1.32)	.19
ER	3.19 (1.31)	3.34 (1.56)	3.53 (1.18)	.01	3.23 (1.44)	3.19 (1.39)	3.35 (1.56)	.14
AM	1.91 (1.32)	2.21 (1.08)	2.12 (1.39)	.27	2.80 (1.55)	2.59 (1.26)	2.64 (1.33)	.09
	Measured after the condition			Measured after the whole experiment				
	Self-Set	COM-B^c^		NR	Self-Set	COM-B^c^		
Appreciation ^b^	5.78 (2.80)	4.96 (2.29)		4.74 (3.04)	7.78 (2.04)	4.22 (2.67)**		

*AM* Amotivation, *ER* External Regulation, *ETI* Easy to initiate, *IM* Intrinsic Motivation, *IR* Identified Regulation, *Irr* Irritation, *Opp* Opportunity, *r* Pearson’s correlation between measured variable and adherence, *SA* Subjective Adherence, *Sat* Satisfaction with adherence, *SE* Self-Empowerment

^a^ the lower the number, the higher the irritation

^b^ appreciation of the reminder type measured from 1 to 10 after a condition, and after the whole experiment

^c^ *in this column means there is a significant difference between conditions

* *p* < .05, ** *p* < .01

### Drop-outs

Thirty people responded to the question why they did not download the app or used it very little. The main reasons were a) unsuccessful in downloading the app, b) problems with the technology, c) inappropriate timing, and d) other reasons. The most prevalent problem was downloading the app. The app was provided via the Google Playstore as a test version, meaning participants had to become part of a Google group, as a result, people had to perform extra steps, which caused problems for people. Furthermore people experienced problems with the technology, e.g. their Android version was too old, or their smartphone broke. Besides technology-related problems, people mentioned that the timing for using the app was not convenient because they were, for example, rehousing or on holiday. Other reasons for dropping out were that people found another solution for their sleeping problem, they did not notice an effect, or they simply forgot to use it.

### Interviews

The interviews indicated that most people were positive about the sleep diary *“[about the diary] It just worked well, it was crisp and clear, I did not have any problems.*” (female, 35 yr). In contrast to the relaxation exercise, which induced more diverse opinions. Some people had a positive attitude towards the relaxation exercise *“ I was surprised that such an easy relaxation exercise helped me that much. I just had to do it every day.”* (female, 39 yr), others thought the exercise was boring *“The relaxation exercise was so-so, especially because every time it was the exact same exercise, so after three days I was bored with it.”* (female, 34 yr), and others prefered to do their own relaxation exercises with which they were already familiar *“I only did the relaxation exercise once or twice, because I already do breathing and meditation exercises. So, the relaxation exercise in the app didn’t have any added value.”* (male, 27 yr). About the reminders participants said that the COM-B reminders were annoying and that the timing was bad *“Well, the reminders came randomly, and then I experienced them as bothersome.”* (female, 54 yr). In general, the self-set reminders were perceived as timed better *“I have the impression that the self-set reminders worked best for me. Those reminders came at the right moments.”* (female, 20 yr), although a few people thought differently *“The self-set reminders were actually not much better than the automatic ones. Both came often at inconvenient times.”*(female, 56 yr). In case people do not get any reminders, they just forget to do an activity *“It was inconvenient when I did not get a reminder, because then I forget to do the activities.”* (female, 34 yr). Interesting was that some people were waiting for the reminder to arrive and perform the activity, even in the No Reminder condition *“When I did not get any reminders, I was kind of waiting for them”* (female, 56 yr).

## Discussion and conclusion

In this paper, we tested reminders in a mobile sleep intervention. On average, participants filled out the sleep diary more often with reminders than without reminders. Also, the relaxation exercise was performed more frequently with reminders compared to the no reminder condition. Both reminders increased adherence thereby supporting the first hypothesis. The results showed that it did not matter which kind of reminder participants received. Support for hypotheses two and three was also obtained, as we found significant mediating effects of self-empowerment and opportunity on adherence for the sleep diary in the regression analysis. However, no support for hypothesis two and three were found regarding mediation effects of self-empowerment and opportunity on adherence to the relaxation exercise. The results of the regression analyses showed a partly mediation, this means that for the self-set reminders, the associated feeling of self-empowerment can explain part of the diary adherence. For the COM-B reminder, one explaining factor is people’s perception that the reminders were given at opportune moments. Besides self-empowerment and opportuneness, different mechanisms are likely at play to why people adhere to the reminders.

The findings show that perceived self-empowerment was higher in the self-set reminder condition than in the COM-B reminder condition, as expected. Opportuneness of the reminders between the two conditions only differed for the relaxation exercise (in opposite direction to expectation), but was not found for the diary entries. The lack in difference in opportuneness has probably been caused by the actual timing of the reminders for the diary, which did not differ that much between the two conditions. In the COM-B condition a diary reminder was sent the first time someone turned on their phone which is most likely shortly after they wake-up, in the self-set condition people probably set the diary reminder a short time after they wake-up as well. So, timing for the diary reminder in the two conditions were most likely very similar.

Exploratory analyses provided more insights in which cases underlying principles, such as self-empowerment and opportuneness, play a role. From the interviews we learned that participants had a negative attitude towards the relaxation exercise. This observation was supported by the relative low appreciation scores given to the relaxation exercise (5.7 on a scale from 1 to 10). This suggests that a positive attitude towards the activity might be a precondition for factors such as self-empowerment and opportuneness to come into play. In case of a negative attitude, which is the case for the relaxation exercise, self-empowerment and opportunity did not explain adherence. A negative attitude probably deters people from exhibiting the targeted behaviour, irrespective of the level of perceived self-empowerment or opportuneness of the moment. Therefore, another sort of trigger might be more suitable for the relaxation exercise.

Several mechanisms have been suggested for why people adhere to reminders. Fogg describes three types of triggers in his behavioural model [7]. If we apply Fogg’s categorization of triggers, the reminders in this experiment mostly resemble signals. We speculate however that the relaxation exercise would benefit more from sparks than from signals, since the appreciation for the relaxation exercise was low. According to Fogg (2009) there are three core motivators that sparks can use: pleasure-pain, hope-fear, and social acceptance-rejection. For the relaxation exercise the reminder could for example emphasize the relaxed state people experience (pleasure) while doing the relaxation exercise. Future research could explore the effect of these different types of reminders.

When examining adherence, it is important to study the participants who dropped-out. By studying the drop-outs insight can be gained about the underlying reasons for not doing something. Approximately half of the participants who downloaded the app only used it for a few days or even did not use it at all. We did our best to contact those people and discover their reasons, which were mainly technical problems, and inappropriate timing to participate in the study. The possible implications of these drop-outs for our results are unknown. It might be the case that more persevere people, or people that already heavily use their phone participated longer in the experiment. Apart from drop-outs, increasing experimental compliance (e.g. filling in weekly questionnaires) also requires attention to obtain the required data set, especially in experiments in the field. In this study approximately 50% of the participants who used the app filled in the questionnaires. Therefore not all participants could be included in the analysis, and some missing data was estimated. Nonetheless, field studies are necessary to ecologically validate mobile interventions, and irreplaceable when studying adherence.

To fully appreciate the findings, it is important to consider the study’s limitations. The main limitation of this study is the implementation of the COM-B reminders. A relative simple algorithm was implemented to detect opportune moments to perform the target behaviour. However, as mentioned before, this might have resulted in diary reminders to occur at similar moments in the two reminder conditions. Furthermore, the algorithm did not anticipate on participants who use their phone minimally. For example, participants might not have received COM-B reminders, if they did not use WiFi. Future research might therefore explore ways to improve the algorithm. Another limitation is the extent of the intervention system. Applications that offer more support, such as cognitive therapy or sleep restriction, might elicit more positive usage attitude. Adherence to reminders might be higher in these applications. On the other hand, applications that offer little support to which people have negative attitudes might also benefit from reminders. For example, adherence to mundane tasks such as hour registration, might improve due to reminders. Next to self-empowerment and opportuneness, other underlying principles, like obligation to employers, probably play a role in such processes.

The main contribution of this study can be summarized by two new insights. First, the study shows that reminders do improve adherence to target behaviours such as keeping a sleep diary and performing relaxation exercises. This is important as adherence has been associated with treatment effect [22]. Second, self-empowerment and opportunity can partly explain why people follow up on reminders and perform the desired activity.

## Appendix

### Measurement overview

**Table:**

Pre-measures	Week 1,2, and 3	Post-measures
Insomnia Severity	Objective adherence	Insomnia Severity Index
GSM usage ability	Subjective adherence	Ability to perform activity
Behavioural intention	Satisfaction with adherence	Score for the reminders
Locus of Control	Easy to initiate	Ranking of the reminders
	if reminder	UTAUT:
	Score/grade for reminder	Utility
	Opportunity	Effort
	Control	Social influence
	Predictability	Facilitating conditions
	Commitment	Attitude
	Motivation Diary	Self-efficacy
	Motivation Relax	Anxiety
	Irritation	Trust
	Remarks*	Behavioural intention

All measures were used in the expectation maximisation algorithm to fill in missing data, except the remarks denoted by *

### Pre-treatment questionnaire

#### GSM ability

I know how to use my smartphone

I know how I can respond to notifications on my smartphone

Other apps that I use, send me reminders sometimes

I regularly set reminders myself using my smartphone

#### Behavioural intention

The theory of planned behaviour states that behavioural intention predicts behaviour [43]. Therefore, behavioural intention (BI) was measured using the six questions below.

I plan to use the app for 3 weeks

I will follow the instructions/advice from the app

I plan to complete my sleep diary every day

I will definitely look at the overview of my sleep data

I am planning to do the relaxation exercise twice a day

If I have a question about the app, I will search for an answer

#### Locus of control

Locus of control (LoC) was measured via a 18-item Dutch questionnaire [44]. A higher internal locus of control has been found to influence diary adherence in an online lifestyle diary [45]. Higher scores indicate a higher external locus of control [44].

### Weekly questionnaire

#### Adherence

**Diary**

How many times did you fill in the diary last week? If you don’t know it exactly, estimate it to your best ability

Why did you not fill in the diary on some days?

I am satisfied with how often I have completed the diary last week.

How many reminders did you get the past week about filling in the diary? If you are not sure, try to estimate it.

**Relaxation**

How many times did you do the relaxation exercise last week? If you don’t know it exactly, estimate it to your best ability

Why did you not do the relaxation exercise on some days?

I am satisfied with how often I have done the relaxation exercise last week.

How many reminders did you get the past week about doing the relaxation exercise? If you are not sure, try to estimate it.

**General**

On a scale from 1 to 10, with 1 being the lowest and 10 being the highest rating. What grade would you give this kind of reminder?

#### Easy to initiate

Easiness to use was measured with four 7-point Likert scale statements. If an activity is hard to integrate in people’s daily life, the probability that people will perform the activity decreases, since people’s behaviour are affected by the principle of least effort [47].

**Diary**

It was hard to make time to fill in the diary

Filling in the diary was kind of a habit for me

**Relaxation**

The relaxation exercises were easy to integrate into my daily routines

I had to put in a lot of effort to not forget to do the relaxation exercise

#### Motivation

For measuring motivation the Situational Motivation Scale (SIMS) [42] was used.

### Weekly Reminder questionnaire

#### Opportunity

**Diary**

The reminders for the diary arrived at inopportune times.

The reminders for the diary were timed well.

I always responded to the reminders of the diary.

*Cronbach’s alpha’s*: Self-set: .443, COM-B: .718

**Relaxation**

The reminders for the relaxation exercise were sent at the right time.

I often dismissed the reminder for the relaxation exercise, because it the time was inappropriate.

Reminders for the relaxation exercises came at times that did not suit me.

*Cronbach’s alpha’s*: Self-set: .776, COM-B: .789

#### Self-Empowerment

**Control - Diary**

I think that I had enough influence on the reminders for the diary

I had enough control over the reminders for the diary

I had no control over the reminders for the diary

*Cronbach’s alpha’s:* Self-set: .944, COM-B: .861

**Control - Relaxation**

I did not have enough control over the reminders for the relaxation exercise

I was in control regarding the reminders for the relaxation exercise

I could not influence the reminders for the relaxation exercise enough

*Cronbach’s alpha’s:* Self-set: .867, COM-B: .923

**Predictability - Diary**

I was not able to predict the reminder times for the diary

Reminders for the diary came unexpectedly

The reminders for the diary came at predictable times

*Cronbach’s alpha’s:* Self-set: .636, COM-B: .711

**Predictability - Relaxation**

I regularly wondered when the reminders for the relaxation exercises would come

The reminders for the relaxation exercises came at moment that I expected them to come

I think the reminders for the relaxation exercises arrived at predictable moments

*Cronbach’s alpha’s:* Self-set: .987, COM-B: .824

**Commitment - Diary**

I felt uncomfortable ignoring the reminders for the diary

When I acted on the reminders for the diary I felt content

I felt guilty when I did not respond to the reminders for the diary

*Cronbach’s alpha’s*: Self-set: .527, COM-B: .797

**Commitment - Relaxation**

I had the feeling I did not stick. to an agreement if I ignored the reminders for the relaxation exercises

I owned it to myself to follow the reminders of the relaxation exercise.

I did not have any trouble ignoring the reminders for the relaxation exercises.

*Cronbach’s alpha’s:* Self-set: .629, COM-B: .616

#### Irritation

Irritation was measured with six 7-point Likert scale statements. If people were irritated by the reminders the chance they will adhere decreases [46].

**Diary**

I got to many reminders for the diary

I appreciated the reminders for the diary

I was annoyed by the reminders for the diary

*Cronbach’s alpha’s:* Self-set: .627, COM-B: .744

**Relaxation**

I think the reminders for the relaxation exercises are nice

I got mad with the reminders of the relaxation exercises

I got to many reminders for the relaxation exercises

*Cronbach’s alpha’s*: Self-set: .688, COM-B: .809

### Final questionnaire – users’ experiences

#### Ability

I found it easy to fill in the diary

I thought it was difficult to perform the relaxation exercise

I totally understood the instructions of the relaxation exercise

I did not understand the instructions of the sleep diary

#### Appreciation

*Reminder preference:* You have received three types of reminders; no reminders; self-set reminders; automatic reminders. Indicate which reminder you preferred. Start on the top with your favourite reminder and end with your least favourite reminder.

*Appreciation reminders:* On a scale from 1 to 10, in which 10 is the highest score, which scores would you give the reminder types below?

- No reminder
- Self-set reminder
- Automatic reminder

*Appreciation app components*:On a scale from 1 to 10, in which 10 is the highest score, which scores would you give the components below?

- The sleep diary
- The relaxation exercise
- The app in total

#### UTAUT

The users’ experiences measure was based on the Unified Theory of Acceptance and Use of Technology (UTAUT) [48]. The Unified Theory of Acceptance and Use of Technology (UTAUT) defines eight concepts that measure technology acceptance and link them to intention and usage, and thereby possibly explain adherence. In addition, trust has been added to the UTAUT model as a predictor, since a lack of trust could negatively influence usage [56, 57]**.**

**Utility/Effect**

With the app I could track my sleep pattern very well

By using the app I could detect problems in my sleep pattern

Using the app improved my daily quality of life

*Cronbach’s alpha:* .712

**Effort**

It was easy for me to figure out how the app worked

The app was easy to use in daily life

The app was complicated, therefore it was hard for me to understand

*Cronbach’s alpha:* .724

**Social Influence**

People who are important to me think I should use the app

My family supported me in using the app

My friends think I should use the app

*Cronbach’s alpha:* .799

**Facilitating Conditions**

The app was not compatible with other products I use

I had the knowledge necessary to use the app

Someone was available for assistance with app difficulties

*Cronbach’s alpha:* .549

**Attitude**

Using the app was a good idea

I liked using the app

I hated using the app

*Cronbach’s alpha:* .751

**Self-efficacy**

I could use the app without any help

Using the app went well as long nothing unexpected happened

If there was no one around to help me, I preferred not to use the app

*Cronbach’s alpha:* .871

**Anxiety**

The app was somewhat intimidating to me

It scared me to think that I could lose information by pressing the wrong button

I think the app could invade my privacy

*Cronbach’s alpha:* .588

**Trust**

I trusted the information the app gave me

I felt distressed using the app

I have confidence in the app working well

*Cronbach’s alpha:* .600

**Behavioural Intention**

I was determined to insert information into to app at the right time

I intended to look at the graphs of my sleep

My intention was to use the app for 3 weeks

*Cronbach’s alpha:* .678

### Interview questions

Why did you use the app?

Did you have any specific goals?

What do you think of the app?

How often did you plan on filling in the diary? And were you successful with that? / How well / often did you fill in the diary? / Every day or did you sometimes not fill it in?

If you filled out the diary when and where you did you usually do it?

Were there certain reasons (obstacles / things / events), why it sometimes did not work out to fill in the diary?

If so, what were those obstacles?

How well did filling in your diary fit in your daily life?

Did the different types of reminders affect whether or not you filled out the diary?

How often did you plan to do the relaxation exercise?

And were you successful with that? / How well / often did you do the relaxation exercise? / Every day or did you sometimes not do it?

If you did the relaxation exercise when and where you did you usually do it?

Were there certain reasons (obstacles / things / events), why it sometimes did not work out to do the relaxation exercise? If so, what were those obstacles?

How well did the relaxation exercise fit in your daily life?

Did the different types of reminders affect whether or not you did the relaxation exercise?

Did you look at your sleep data? If so, when did you look at it? What exactly did you want to know / see? Did you find it? What do you think about the way the data is displayed?

To what extent was the app beneficial and effective?

- What effect had the diary in your sleep?
- What effect had the relaxation exercise on your sleep?
- What kind of effect did the app have on yourself?
- Did the app had an effect on something else in your life?

Were there any irritations regarding the app?

Did it cause irritation when you received reminder, or did you thought it was fine? What did you think about the reminders? What kind of reminder irritated you the most?

What would you like to see improved in the app? And what else?

What properties would you like to add to the app?

What can be added to the app, so you would adhere (even) longer or better?

Would you recommend the App to others? Why?/ Why not?

What did you think of the experiment itself, not the app, but everything else, such as emails, surveys, downloading, etc.?

Are there other things you want to say about the app that I have not covered?

Do you have any other comments or questions?

## Footnotes

1 Only the top-10 apps in the Netherlands triggered a reminder: Whatsapp, Facebook, Candy Crush, Nu.nl, Twitter, NOS, Wordfeud, Minecraft, Wheres My Water, Ruzzle, Browser.
